# Supplementary material for: Health and science-related disinformation on COVID-19: A content analysis of hoaxes identified by fact-checkers in Spain
Source: PLoS One. 2022 Apr 13;17(4):e0265995. doi: 10.1371/journal.pone.0265995 (PMC9007356; doi:10.1371/journal.pone.0265995)
Supplement: S1 Appendix — Coding criteria and variables developed to classify the hoaxes. (DOCX) [file pone.0265995.s001.docx]

**Supporting information 1**

**Codebook**

1. **Hoax ID**: Number to identify each hoax.
2. **Date**: March 11 to June 10, 2020, inclusive
3. **Verification title**: Verification title given by the fact-checking platform
4. **Verification URL**: URL for verification.
5. **Checker**: fact-checking platform

I. Maldita

II. Newtral

III. EFE Verifica

1. **Main platform used to spread the hoax mentioned in the verification (More than one may be selected)**

I. Social networks (not specified)

II. Media

III. Twitter

IV. Facebook

V. WhatsApp

VI. Instagram

VII. YouTube

VIII. Other: specify

1. **Format (More than one may be selected)**

I. Text (including photos of the text).

II. Photo

III. Audio

IV. Video

V. Other

1. **Topic 1 (Only one may be selected)**

Science/health: The hoax concerns scientific or health-related topics.

II. Politics: The hoax relates to political management or actions of politicians.

III. Other: specify

1. **Topic 2. If it relates to science/health (Only one may be selected)**
2. Scientific policy
3. Advice issued to the public (to combat or prevent diseases)
4. Scientific research (e.g., origin of the virus, fatality rate, vaccines, treatments, and transmissibility)
5. Other (specify)
6. **Topic 3. If related to scientific research (Only one may be selected)**
7. Origin of the virus
8. Vaccines
9. Transmissibility
10. Treatments
11. Fatality rate
12. Other (specify)
13. **Source (1)**
14. Anonymous: Unknown or concealed source
15. Real: genuine source
16. Spoofed: Source fraudulently disguised as legitimate.

IV. Fictitious: fake, invented source.

1. **Source (2). If not anonymous, type of real source (Only one may be selected)**
2. Healthcare/science: Healthcare personnel, scientists, or members of a healthcare/scientific institution
3. Government: Holds a position at any level (local, regional, national, international).
4. Professionals
5. Members of the public: Citizens.
6. Political: Belonging to a political party without holding a government position
7. Business companies or organizations
8. Other
9. **Source (3). If the source is health care/science (12) (only one may be selected)**
10. Researcher
11. Healthcare professionals (e.g., doctors and nurses)
12. International scientific organization (spokesperson or representative)
13. National scientific organization (spokesperson or representative)
14. Other (specify)
15. **Geographical scope**
16. Local refers to a specific autonomous community or lower administrative level.
17. National
18. International: A foreign country is explicitly mentioned in the location of the event or in the dissemination of the hoax
19. Not applicable or not specified
20. **Type of hoax**
21. Deception: Misleading use of information to frame a problem or an individual
22. Decontextualization: Truthful content with false contextual information (e.g., location, time).
23. Exaggeration: Intentionally exaggerated reality based on partially true information.
24. Joke/parody: Not intended to cause harm, but with the potential to deceive.
