# Supplementary material for: Health and science-related disinformation on COVID-19: A content analysis of hoaxes identified by fact-checkers in Spain
Source: PLoS One. 2022 Apr 13;17(4):e0265995. doi: 10.1371/journal.pone.0265995 (PMC9007356; doi:10.1371/journal.pone.0265995)
Supplement: S2 Appendix — Cohen’s kappa test was used for inter-coder reliability and the chi-square test was used for significance between variables. Coding dataset. Accessible on Zenodo. DOI:10.5281/zenodo.4895047. https://zenodo.org/record/4895047#.YLfDrLczbct. (DOCX) [file pone.0265995.s002.docx]

**Supporting information 2**

**Statistical analysis**

**Inter-coder reliability**

An inter-coder reliability test was conducted. It consisted of the independent and blind coding, by two different peopleof 515 items for the first analysis of Topic 1, and 126 items for the rest of the analysis. This is a representative sample of the initial universe of 187 items (50% heterogeneity, 5% error margin, and 95% confidence level).

The agreement was tested using Cohen’s kappa test for the parallel blind codification of each variable, resulting in an optimal level of agreement for all variables. Here are the results:

- 1. **Topic 1.** Cases: 533; agreement: 515; options: 3; **Cohen’s kappa: 0.96**
  2. **Topic 2 (if science/health):** Cases: 126; agreement: 122; options: 4; **Cohen’s kappa: 0.95**
  3. **Topic 3 (scientific research).** Cases: 126; agreement: 123; options: 5; **Cohen’s kappa: 0.97**
  4. **Source 1.** Cases: 126; agreement: 125; options: 4; **Cohen’s kappa: 0.98**
  5. **Source 2 (Non-anonymous).** Cases: 126; agreement: 121; options: 7; **Cohen’s kappa: 0.95**
  6. **Source 3 (health/scientific).** Cases: 126; agreement: 125; options: 5; **Cohen’s kappa: 0.99**
  7. **Geographical scope.** Cases: 126; agreement: 126; options: 4; **Cohen’s kappa: 1**
  8. **Type of hoax.** Cases: 126; agreement: 124; options: 4; **Cohen’s kappa: 0.97**

**Test of significance**

To analyze the relationship between the two categorical variables, we conducted a chi-squared test to compare the significance of the relationship between them.

**General contents of the hoaxes by month (Table 3)**

Results: Chi-square = 2,413; df=4; p-tail=0,660. Therefore, there was no evidence of a relationship between the two variables.

| **Chi-Square Tests** | | | |
| --- | --- | --- | --- |
|  | Value | df | Asymptotic Significance (two-sided) |
| Pearson’s Chi-Square | 2.413^a^ | 4 | .660 |
| Likelihood Ration | 2.438 | 4 | .656 |
| Linear-by-Linear Association | .997 | 1 | .318 |
| N of Valid Cases | 533 |  |  |

*Note: *p<.1; **p<.05; ***p<.01*

**Types of hoax according to format (Table 8)**

Since some hoaxes employed more than one format, before testing the possible relationship between the two variables, it was necessary to recode the information as follows:

|  | Frequency | Percentage | Valid percentage | Accumulated percentage |
| --- | --- | --- | --- | --- |
| Text | 264 | 49.5 | 49.5 | 49.5 |
| Photo | 12 | 2.3 | 2.3 | 51.8 |
| Audio | 18 | 3.4 | 3.4 | 55.2 |
| Video | 44 | 8.3 | 8.3 | 63.4 |
| Text and audio | 9 | 1.7 | 1.7 | 65.1 |
| Text and video | 15 | 2.8 | 2.8 | 67.9 |
| Photo and video | 2 | .4 | .4 | 68.3 |
| Audio and video | 1 | .2 | .2 | 68.5 |
| Text, photo and audio | 2 | .4 | .4 | 68.9 |
| Text, photo and video | 3 | .6 | .6 | 69.4 |
| Other formats | 163 | 30.6 | 30.6 | 100.0 |
| Total | 533 | 100.0 | 100.0 |  |

Results: chi-square= 45,494**; df=30; p-tail=0,035. Therefore, there is evidence of a significant relationship between these two variables.

| **Chi-Squared Tests** | | | |
| --- | --- | --- | --- |
|  | Value | df | Asymptotic Significance (two-sided) |
| Pearson’s Chi-Square | 45.494^a^ | 30 | .035 |
| Likelihood Ration | 47.576 | 30 | .022 |
| Linear-by-Linear Association | .712 | 1 | .399 |
| N of Valid Cases | 393 |  |  |

*Note: *p<.1; **p<.05; ***p<.01*

**Types of hoax and geographical location (Table 9)**

Results: Chi-square =7,256; df=9; p-tail=0,6610. Therefore, there was no evidence of a relationship between these two variables.

| **Chi-Squared Tests** | | | |
| --- | --- | --- | --- |
|  | Value | df | Asymptotic Significance (two-sided) |
| Pearson’s Chi-Square | 7.256^a^ | 9 | .610 |
| Likelihood Ration | 9.822 | 9 | .365 |
| Linear-by-Linear Association | .222 | 1 | .637 |
| N of Valid Cases | 392 |  |  |

*Note: *p<.1; **p<.05; ***p<.01*

**Non-anonymous source types by hoax type (Table 11)**

Results: chi square=27,090*; df=18; p-tail=0,077. Therefore, a relationship exists between the two variables.

| **Chi-Squared Tests** | | | |
| --- | --- | --- | --- |
|  | Value | df | Asymptotic Significance (two-sided) |
| Pearson’s Chi-Square | 27.090^a^ | 18 | .077 |
| Likelihood Ration | 26.749 | 18 | .084 |
| Linear-by-Linear Association | .052 | 1 | .819 |
| N of Valid Cases | 270 |  |  |

*Note: *p<.1; **p<.05; ***p<.01*
