# Supplementary material for: Health and science-related disinformation on COVID-19: A content analysis of hoaxes identified by fact-checkers in Spain
Source: PLoS One. 2022 Apr 13;17(4):e0265995. doi: 10.1371/journal.pone.0265995 (PMC9007356; doi:10.1371/journal.pone.0265995)
Supplement: Attachment — Submitted filename: Response to reviewers.docx [file pone.0265995.s003.docx]

| **Reviewers’ comments** | **Responses** |
| --- | --- |
| **Abstract** |  |
| The Abstract presents the results in "The "prototypical hoax"..." sentence as a hodge-podge of observations that are difficult to contextualize, and understand their importance.  Abstract - "importance of time" should be "importance of timing"? | The abstract has been amended. |
| **Introduction, focus** |  |
| Clarify research goals  The paper suffers from a vague motivation, from the introduction, to the presentation of results, and conclusion  It could be strengthened by **motivating the labeling exercise** by a particular task (say, automated hoax detection, or science decontextualization monitor),  I believe **considering the "why is this interesting" question** by the authors may make this observational study much more interesting.  **a particular theory** (ex: pre-publishing scientific news results in misinformation),  or **emphasis on the peculiarity of the Spanish** situation and its interaction with the ongoing events there and abroad. | The aim of our study has been clarified. We keep the original aim of formulating a typology of hoaxes but we argue that this can help to develop future systems for automated detection of hoaxes (l. 86):  *Previous research on COVID-19 information indicates that automated detection can reach high levels of accuracy in detecting credible and non-credible tweets [5]. Therefore, considering that category frequencies are useful for automated numerical analysis [6], we argue that a typology of hoaxes, based on the relationship among different variables related to scientific content, can help develop future systems for automated detection of hoaxes about science and health.*  Our sampling method (selection of hoaxes debunked by fact-checkers) does allow us to know which hoaxes originated from pre-prints. In the conclusion section, we suggest this connection as desirable focus for future research (l. 672):  *Our study also suggests that preprints may be a prominent source of hoaxes. A more profound analysis of that relationship surpasses the aim and scope of our research since our sampling method does not allow the establishment of hoaxes originating from a preprint. However, the connection between preprints and hoaxes is a desirable focus for future research.*  We now explain that although the case of Spain may not be different to that of other countries, it is a perfect case for our study (l. 68):  *Spain was hit hard by the pandemic and suffered a high percentage of infections and deaths [4]. On March 1515 March, 2020, the government announced a state of alarm, which involved a national lockdown that lasted until June 2121 June. Although the situation in Spain during the first months of the pandemic may not differ significantly from that in other countries, the fact that it was highly affected and subdued to a national lockdown created a crisis situation in which citizens were eager to receive information and to use social media. In this context, Spain represented a perfect case for our research.* |
| In the Introduction, the authors claim that the "characteristics of these hoaxes and the forms they take have not yet been brought to light", such as [1] concerning India, and I'm sure many more  1] Akbar, Syeda Zainab, et al. "Misinformation as a Window into Prejudice: COVID-19 and the Information Environment in India." Proceedings of the ACM on Human-Computer Interaction 4.CSCW3 (2021): 1-28. | We have nuanced this idea (l. 61):  *However, the relationship between scientific information and the characteristics of these hoaxes has not yet been elucidated.* |
| More solid and wide literature review | We have conducted a more solid literature review (l. 190-245). |
| Re. Salaverría et al.  In light of the related article, please cite and discuss the above mentioned related paper in the body of the manuscript (introduction, methods if overlap, and discussion). In particular, please discuss how your research contributes to the basis of academic knowledge in light of the above mentioned publication. Please bear in mind that our publication criteria state " If a submitted study replicates or is very similar to previous work, authors must provide a sound scientific rationale for the submitted work and clearly reference and discuss the existing literature. Submissions that replicate or are derivative of existing work will likely be rejected if authors do not provide adequate justification. | We have clarified the relationship with the previous study in the introduction (l. 242):  *Finally, our previous research, based on hoaxes debunked by fact-checkers in Spain, found that one-third of the false information about the pandemic contained falsehoods about scientific and health-related matters [2]. This provides a relevant starting point for the present research.*  Furthermore, in the methodology section, we explain this (l. 283):  *This methodology was similar to that used in our previous study. In both studies, we used the same three fact-checking platforms to select the sample, and a similar codebook for content analysis. However, in the second study, we enlarged the sample from one month to three months, and we focused specifically on health- and science-related hoaxes to dig deeply into their characteristics and types of hoaxes in this topic category.* |
| **Methodology-results** |  |
| I commend the authors on coming up with a coding schema that resulted in a higher inter-annotator agreement, giving me confidence that the labels applied in this study are somehow valid. | We have conducted a more solid intercoder reliability test (Cohen’s kappa test), resulting in an optimal level of agreement (l. 315):  *Once the codebook was established, an intercoder reliability test was conducted. It consisted of the double and blind codification of 126 items, a representative sample of the initial universe of 187 items (50% of heterogeneity, 5% error margin, 95% level of confidence).*  *The agreement was tested through a Cohen’s kappa test for the parallel blind codification of each variable, resulting in an optimal level of agreement for all the variables: Topic 1, 0,96; Topic 2 , 0.95; Topic 3, 0.97; Source 1, 0.98; Source 2, 0.95; Source 3, 0.99; Geographical scope, 1; Type of hoax, 0.97 (see S2 Appendix: Statistical analysis).* |
| The authors need to show statistical testing results when claiming comparison, such as on line 341, especially since the final number of documents examined (n) can shrink quite a bit. In the end, this is a sample of all potential misinformation out there -- if we are comparing, say, 5 to 6 documents, is the strength of this difference enough to claim it can be generalized? | We have conducted a test of statistical significance (l. 324):  *A chi-square test was used to analyze the relationship between two categorical variables, in order to comparecontrast the significance of the relationship between both of them. The results are indicated in the manuscript where appropriate and are included in more detail in S2 Appendix: Statistical analysis Apendix*. |
| **Discussion** |  |
| The analysis presents some interesting insights. For instance, the fact that these hoaxes are often based on "decontextualization" and that this often happens around healthcare/scientific sources.  In fact, the authors state several times that the pace of scientific publishing picked up so much that it's likely that even the publishers "were notable to process them properly". The authors provide several examples of poor-quality and retracted articles that made a splash on social media. It would be very interesting to see a systematic analysis of whether pre-publishing articles resulted in retraction and misinformation, instead of seeing this anecdotal evidence. | We have added an explanation about the meaning of decontextualization (l. 447):  *These were followed by hoaxes based on decontextualization (22.9%), in which the information was placed in a false context. This includes several types of wrong attributions, such as geographical or chronological displacement For example, one of the debunked hoaxes stated that, during the pandemic, the Spanish Prime Minister would have a personal medical team of 14 people as a special measure against the coronavirus [48]. In fact, this is a regular medical team assisting the Prime Minister, not one specially created to protect him during the pandemic.*  Besides, we have added this paragraph to the conclusion and limitations section:  *Decontextualization plays a relevant role in health and science-related hoaxes, probably due to the fact that they are easy to produce and at the same time are credible, due to the fact that they are partially true. Our sample does not allow for a deeper analysis of the differential characteristics of hoaxes based on decontextualization, but this could be a relevant focus for future research.* |
| **Conclussion and limitations** |  |
| The fact that lots of these hoaxes were found on social media channels is emphasized by the authors as a major finding in the paper. However, this is largely affected by the article selection policy of the fact-checking websites used in this study. I am not sure about the Spanish mass media, but in the US (with which I am more familiar), plenty of falsehoods are promoted on the television and radio -- media which still have a huge audience, possibly in demographics only somewhat overlapping with that captured by the current study. I would recommend the authors contextualize their findings in this methodological limitation. | We have acknowledged this limitation in the methodology section (l. 269):  *Fact checks facilitate a reliable source of research, as they allow the identification of pieces of misinformation in a timely manner. However, this method has its limitations, as fact-checkers cannot address all the misinformation that is spread in a particular period of time, and they may have selection biases [44].*  We have also added this paragraph to the results section (l. 385):  *These results are affected by our sample selection method, which is limited to hoaxes debunked by the three aforementioned fact-checking organizations. Therefore, we do not have a sample of all the hoaxes that were published in Spain during this period of time, but rather a sample of a particular selection made by the three organizations. The fact-checkers follow several methods to identify the hoaxes. In some cases, they check the information directly sent to them from the audience. In other cases, they select information previously published by the news media*. |
| Another limitation is the paper's scope - Spain - making the insights highly localized. This is, of course, also a strength, providing points of view outside US-centric research that is so popular.  However, the authors fail to really juxtapose their findings to those in other countries, or strongly contextualize the findings in the peculiarities of the Spanish COVID situation at the time.  This is another area where the authors could strengthen the paper. For instance, a time visualization could provide readers insights into when most hoaxes happened, and with what major events they were associated.  How the political situation may have differed from that of other countries, or influenced by outside political forces (such as Trump's push for hydroxychloroquine). From where the sources of information came (inside or outside Spain), etc. | We acknowledge this limitation in the conclusion-limitations section (l. 687):  *The main limitation of our study is that it analyzed only hoaxes identified by three fact-checking organizations in one country and spread during the first three months of the pandemic only.*  We argue that the situation in Spain may not be different to that of other countries, but it is still a perfect case for our study (l. 70):  Although the situation in Spain during the *first months of the pandemic may not differ significantly from that in other countries, the fact that it was highly affected and subdued to a national lockdown created a crisis situation in which citizens were eager to receive information and to use social media. In this context, Spain represented the perfect case for our study.*  A higher percentage of hoaxes were debunked by the fact-checkers during the first month of the pandemic. However, there is no significantly higher percentage of hoaxes about science and health in any of the three months, as we explain in this paragraph (l. 357):  *With respect to the period under study, the highest number of hoaxes was observed in the first month. From the time the WHO announced the pandemic on March 11 to April 10, 293 hoaxes were recorded, that is, 54.97% of the total. Of these, just over one-third (109) corresponded to “science and health” hoaxes (Table 3). However, the distribution of content of hoaxes by month is not statistically significant (chi-square = 2,413; df = 4 y p-tail>0,05), which means there was not a specific month in which a significantly higher proportion of hoaxes about “science and health” was spread.*  In the discussion section (l. 536), we explain this:  *Almost half of all hoaxes (43.7%), including 54.9% of science and health-related hoaxes, were spread in the first month of the pandemic. This suggests that, in the early days, the strong public interest in accessing information to adapt to a novel situation combined with a lack of information gave rise to numerous hoaxes. However, hoaxes about science and health continued to spread consistently during the three sampling months, which makes it difficult to establish a causal relationship between the diffusion of misinformation and specific events beyond the beginning of the pandemic that coincided with the first few weeks of the lock-down in Spain.* |
| **Other comments** |  |
| Provide dataset |  |
| Please include additional information regarding the data extraction tool used in the study and ensure that you have provided sufficient details that others could replicate the analyses. For instance, if you developed a data extraction tool as part of this study and it is not under a copyright more restrictive than CC-BY, please include a copy, in both the original language and English, as Supporting Information, or include a citation if it has been published previously. | We have now explained this (l. 275):  *The sample was manually selected, checking the websites of the three organizations, and compiling all the items related to COVID-19. No automated or specific data extraction tools were not used.* |
| Include captions for your Supporting Information files at the end of your manuscript, and update any in-text citations to match accordingly. | We have included captions (l. 907) |
| We suggest you thoroughly copyedit your manuscript for language usage, spelling, and grammar. If you do not know anyone who can help you do this, you may wish toconsider employing a professional scientific editing service. | The manuscript have been professionally copyedited by Editage. |
