# Supplementary material for: Health and science-related disinformation on COVID-19: A content analysis of hoaxes identified by fact-checkers in Spain
Source: PLoS One. 2022 Apr 13;17(4):e0265995. doi: 10.1371/journal.pone.0265995 (PMC9007356; doi:10.1371/journal.pone.0265995)
Supplement: Attachment — Submitted filename: Response to reviewers.docx [file pone.0265995.s004.docx]

**Health and science-related misinformation on COVID-19: A content analysis of hoaxes identified by fact-checkers in Spain
Review 2 (20-10-2021)
Response to reviewers**

| **Reviewer comments** | **Responses** |
| --- | --- |
| The result is descriptive and lacks knowledge discovery, it would be really nice if the author could consider more comparison or in-depth analysis. | Our results provide knowledge that goes beyond the specific case study. As we explain the conclusion, we have   1. Identified the most common characteristics of health and science related hoaxes.   *These characteristics provide relevant information that can be used as a basis for future research. They can also contribute to a better understanding of how disinformation is spread to the public and, therefore, can help to improve media literacy actions about health and science* (p. 32).   2. Formulated a typology of this type of hoaxes, according to their relationship to scientific information.   T*his typology can work as a basis for future research and can help to develop systems for automated detection of health and science- related hoaxes* (p. 34). |
| The statistical analysis has not been performed appropriately and rigorously. For example, for chi-square, which should be used to compare two variable, seems to  be used to compare multiple variables at one time (or haven’t been reported properly) as shown in one example below (line 451-453), and p value should not be  reported as “p-tail<0,05”:"A significant difference between formats was that audio clips were used more frequently in the case of exaggerations (20.0%) (Table 8)  (chi-square= 45,494**; df=30 ; p-tail<0,05). | The chi-square test was used to compare two variables but the results were not reported properly. We have now amended this in the main text and Appendix 2. |
| The authors use content analysis with Codebook by two coders, there are 3 topics however, in result, only four types are listed, which doesn't match:  Healthcare/science,Healthcare/science,Member of the public, Business.（Table 11. Non-anonymous source types by hoax type.) | We have amended the manuscript in order to differentiate correctly the four types of hoaxes (deception, decontextualization, exaggeration, and joke/parody.) and the three topics of Science and health-related hoaxes (Scientific research, Scientific policy and health management, Erroneous advice issued to the public):  *The classification of hoax content revealed relatively similar frequencies among the three main topics* (p. 17).  *Healthcare/scientific sources predominated in three of the four hoax type*s (p. 24). |
| I would suggest this research to distinguish the difference between hoaxes, rumors, misinformation and fake news. There have been many studies on  misinformation, rumors and fake news, so why choose hoaxes only? Comparison between hoaxes and misinformation and further analysis could be added to  enhance the importance of the article. | We have clarified this in the text. As the manuscript indicates, the conceptualization regarding the forms of falsehood in publicly disseminated information is based on a “theoretical distinction between disinformation and misinformation”. The first concept refers to deliberate deception, while the second covers inadvertent falsehoods. Ultimately, these two categories distinguish between lying (voluntary) and error (involuntary).  Within these two general categories there are multiple concrete expressions. Specifically, research has explored modalities such as conspiracy theories (Craft et al. 2017), rumors (Alkhodair, 2020) and hoaxes (Braun & Eklund, 2019). In the field of journalism and the media, the so-called “fake” (Tandoc et al., 2021) or “false” (Andı & Akesson, 2020) news have also been widely investigated.  In our study, we have opted for using the concept of “hoaxes”, because the falsehoods investigated do not correspond only to content disseminated in news media and because, according to the existing literature, it is a concept that designates deliberate falsehoods targeted to the general public through any communication channel.  References  Alkhodair, S. A., Ding, S. H., Fung, B. C., & Liu, J. (2020). Detecting breaking news rumors of emerging topics in social media. *Information Processing & Management*, 57(2), 102018.  Andı, S., & Akesson, J. (2020). Nudging Away False News: Evidence from a Social Norms Experiment. *Digital Journalism*, 9(1), 106-125.  Braun, J. A., & Eklund, J. L. (2019). Fake news, real money: Ad tech platforms, profit-driven hoaxes, and the business of journalism. *Digital Journalism*, 7(1), 1-21.  Craft, S., Ashley, S., & Maksl, A. (2017). News media literacy and conspiracy theory endorsement. *Communication and the Public*, 2(4), 388-401.  Tandoc Jr, E. C., Thomas, R. J., & Bishop, L. (2021). What is (fake) news? Analyzing news values (and more) in fake stories. *Media and Communication*, 9(1), 110-119. |
| In the introduction, the author claim to" establish a classification system that can be used to explain the narrative mechanisms that underpin the credibility of this  information "(line. 58,59), however, instead of building up a system, this paper demostrates a descriptive analysis on one case only. | We have amended this phrase and clarified the research objectives in the introduction (p. 5):  *The overall objective of this study is to analyze the science and health related hoaxes about COVID-19 that were spread during the pandemic. More specifically we aim to (1) identify the characteristics of form and content and the platforms used to spread science and health related hoaxes; and (2) formulate a typology that can be used to classify the different types of hoaxes, according to its connection with scientific information.* |
