# Supplementary material for: Health and science-related disinformation on COVID-19: A content analysis of hoaxes identified by fact-checkers in Spain
Source: PLoS One. 2022 Apr 13;17(4):e0265995. doi: 10.1371/journal.pone.0265995 (PMC9007356; doi:10.1371/journal.pone.0265995)
Supplement: Attachment — Submitted filename: Reviewers Comments. PONE-D-20-35335R2.docx [file pone.0265995.s005.docx]

**PONE-D-20-35335R2**

**Health and science-related misinformation on COVID-19. A content analysis of hoaxes identified by fact checkers in Spain**

**Reviewer’s Comments**

**General comments**

I provide the following suggestions to help the authors improve the manuscript.

I believe that the manuscript would benefit greatly from a rigorously executed reduction in its length. It is overly wordy and this impedes readability. It requires more concise, succinct writing and the removal of repetition. A more logical flow is required, especially in the ‘Introduction’ and the ‘Conclusions’ sections. These changes are also necessary to bring the manuscript to publishable standard, and to keep the reader engaged.

I have not commented on the statistical tests or calculations as this has been done by another reviewer. I have, however, checked calculations in all of the tables. There are multiple, minor errors in the percentage calculations in most of the tables. These occur at the first or second decimal place. They are noted, below.

I encourage the authors to re-work the manuscript to make it shorter, more coherent and more readable, in order to convey their research more compellingly.

This is an interesting and comprehensive manuscript. It reports valuable research on a topic that is very important to society, internationally.

The use of Spain as a case example, and informed by the Spanish and international literatures and experiences, is most appropriate. The authors have developed a useful typology for use in future research. This is commendable.

**Editorial comments**

When describing the study – throughout the manuscript – please use the past tense consistently. This should have been done/corrected by the English language editing service (referred to in ‘Acknowledgements’, line 690).

Similarly, the English language editing service should have removed ‘However’ and ‘Although’ from the beginning of sentences; this sloppy syntax reads poorly in a prestigious international journal.

**Introduction**

The ‘Introduction’, including the literature review component, would benefit substantially from re-organisation and reduction in length, including the removal of repetition. I strongly recommend that this be done.

Para 2: The aim and objectives should be moved to the end of the ‘Introduction’ and placed immediately before the ‘Materials and Methods’ section.

The aim is the broad, overarching focus of a study. It appears to be stated correctly in the Abstract; however, in para 2 of the ‘Introduction’, it is erroneously stated as the ‘objective’. Please amend this.

The objectives should fall under the aim and are the more specific foci of the study, i.e. ‘… to (1) identify the characteristics of form … ; and (2) formulate a typology … .’ I suggest that they be stated as such, i.e. specifically as objectives, in both the ‘Abstract’ and in the Introduction and anywhere else they are mentioned, e.g. ‘Discussion’.

Para 3: This pre-empts the ‘Method’. This para should be removed from the ‘Introduction’ as these facts belong in the ‘Materials and Method’ section.

Para 5, line 80: The aim is repeated. Please remove.

Para 6, beginning line 86: Sentence 1 should be moved to the ‘Conclusions’.

Para 8, line 101: The reference to Ulysses is extraneous. I suggest that the sentence be removed.

Paras 10-12: The definitions of key terms such as ‘disinformation’, ’hoaxes’, etc., are sound and appropriate.

Para 13: Sentence 1 needs to be re-worked for clarity, ‘… much research. This has reflected a wide range of ….. ‘.

Para 14: ‘Misinformation’ is discussed; however, the following sub-section is headed, and is about, ‘misinformation’.

Paras 19-21: These belong in the early part of the Introduction, i.e. as background. They could usefully be reduced in length.

Para 26, lines 244-6: This belongs in the Limitations (sub-section of the ‘Discussion’).

Para 27: Split the first sentence into two sentences (lines 247-250); the sentence is currently too confusing for the reader. The last phrase could be re-worked to acknowledge the source, e.g. something along the lines of ‘Evidence suggests that conspiratorial claims are … .’

Suggestion for some parts of the literature review: The authors have obtained a wide range of very useful sources to provide Spanish and international context for their study. The items cited in the literature review should include mention of the geographical location, i.e. where the studies were conducted. This detail is included for some articles, but not others. The geographical location, even if the study was conducted in a large region, or was ‘multi-country’ study, is important contextually.

**Materials and methods**

The structure of this section requires considerable re-work.

Para 1, beginning line 256: Remove this para. It is not relevant here.

I suggest that the authors include the sub-heading ‘Study design’ and mention the fact that they have used qualitative method and refer to a relevant source (such as Hsieh H-F and Shannon SE [2005] Three approaches to qualitative content analysis. *Qualitative Health Research* 15(9): 1277-1288).

Para 3: This should have a sub-heading ‘Data collection’. The repetition should be removed.

Para 4, lines 273-276: Move to the ‘Limitations’ sub-section at the end of the ‘Discussion’.

Para 4, lines 276-278: Note that part of this is repeated from earlier. Make sure that the study period, and its relationship to the WHO declaration of the pandemic, are only mentioned in the Method – and not in the ‘Introduction’.

Para 5, lines 287-292: The method used in the previous study is irrelevant to the current study. Remove these sentences.

Para 5, lines 292 et seq: State the authors of the previous study.

Para 5: Were the two coders members of the research team? If so, include their initials in brackets in the final version of the manuscript. This level of detail is important when reporting qualitative research. Similarly, the engagement of the researchers with the data at this level is very important in qualitative research.

Para 6: The list of variables might be inserted into a box, for presentation purposes.

**Results**

All tables: Line up ones under ones, tens under tens, etc.

Include footnote explaining rounding in percentage column (where this has occurred).

All paras: Take care to remove extraneous phrases, e.g. para 2, line 344 ‘At the same time,’.

Table 2: This seems to lack order (L. column), e.g. alphabetical order according to category or size of ‘*n*’ for each category. The caption refers to “other”, yet there is also a row labelled “other”. This is confusing, i.e. there is a category of ‘other’ in the ‘other category’.

Furthermore, it is unusual to list ‘Other’ (the residual category) in the first row. Normally this is shown in the last row, above the Total row. It would be useful to include a footnote to explain what ‘other’ contains (the *n*=3 row).

Table 2, Row 1 (‘Other’): Percentage statistic should be 1.76 (not 1.77). Please correct in the Table, and in any references that happen to be in the text.

Para 4: I have mentioned this above, but please remove ‘However’ from the start of sentences, e.g. lines 368 and repeated in the following sentence beginning on line 371.

Para 6, beginning line 381, first two sentences: These belong in the ‘Discussion’. The ‘Results’ section should only include the actual results, and not the researchers’ interpretations and discussion of these.

Para 6, lines 385-387: These sentences probably belong in the Discussion.

Table 4: The statistics in the percentage column are incorrect. Please correct in the Table, and in any references that happen to be in the text.

Table 5: There are minor errors in each statistic in the percentage column. Please correct in the Table, and in any references that happen to be in the text.

Table 6: There are minor errors in most of the statistics in the percentage column. Please correct in the Table, and in any references that happen to be in the text.

Para 13, line 455: Use full word, ‘advertisement’.

Table 7: There are minor errors in most of the statistics in the percentage column. Please correct in the Table, and in any references that happen to be in the text.

Table 8:

There are minor errors in all statistics in the ‘Deception’ percentage column. Please correct in the Table, and in any references that happen to be in the text.

There are minor errors in most statistics in the ‘Decontextualization’ percentage column. Please correct in the Table, and in any references that happen to be in the text.

There are minor errors in most statistics in the ‘Exaggeration’ percentage column. Please correct in the Table, and in any references that happen to be in the text.

There are minor errors in all statistics in the ‘Total’ percentage column. Please correct in the Table, and in any references that happen to be in the text.

Para 16, lines 486-7: Move this definition forward, to earlier in this paragraph.

Table 9:

There are minor errors in all statistics in the ‘Deception’ percentage column. Please correct in the Table, and in any references that happen to be in the text.

There are minor errors in all statistics in the ‘Decontextualization’ percentage column. Please correct in the Table, and in any references that happen to be in the text.

There are minor errors in all statistics in the ‘Exaggeration’ percentage column. Please correct in the Table, and in any references that happen to be in the text.

There are errors in statistics in the ‘Total’ percentage column. Please correct in the Table, and in any references that happen to be in the text.

Para 18, line 499: Li Wenliang was an ophthalmologist (i.e. a specialist eye doctor); he should be described as a doctor, not a scientist.

Table 11:

There are minor errors in all statistics in the ‘Deception’ percentage column. Please correct in the Table, and in any references that happen to be in the text.

There are minor errors in some statistics in the ‘Decontextualization’ percentage column. Please correct in the Table, and in any references that happen to be in the text.

**Discussion**

Para 5, lines 568-9: It may be advisable to omit the descriptor of Didier Raoult as ‘eccentric’. This could be legally problematic for the journal and its Editors. The following would be suitable wording: ‘The French microbiologist …… ‘. The fact that Raoult was an advisor to the French government suggests that he is ‘well-known’. The rest of this paragraph could be condensed a little.

As mentioned above, include a sub-section titled ‘Limitations’ as the last part of the ‘Discussion.

**Conclusion**

Para 1, beginning line 629: This should be ‘objective’ and not ‘goal’.

Tables 12 and 13 are excellent summary tables. They present findings and, as such, essentially belong in the ‘Results’; however, given their summary nature, they could be integrated into the ‘Discussion’. Technically, they do not belong in the ‘Conclusions’.

Para 7, lines 665—7, second sentence: This comment belongs in the ‘Limitations’ sub-section. The present paragraph could be amended to: ‘Our findings also suggested that preprints, which were beyond the scope of our study, may be a prominent source of hoaxes. The connection between preprints and hoaxes presents a desirable focus for future research.’

Para 11, beginning line 679: Move sentence 1 (limitation) to the ‘Limitations’ sub-section of the Discussion.

--------------------------
