# Supplementary material for: Health and science-related disinformation on COVID-19: A content analysis of hoaxes identified by fact-checkers in Spain
Source: PLoS One. 2022 Apr 13;17(4):e0265995. doi: 10.1371/journal.pone.0265995 (PMC9007356; doi:10.1371/journal.pone.0265995)
Supplement: Attachment — Submitted filename: Reviewer 3. Final Comments and Recommendation. PONE-D-20-35335R2.docx [file pone.0265995.s007.docx]

**PONE-D-20-35335R2**

**Health and science-related misinformation on COVID-19. A content analysis of hoaxes identified by fact checkers in Spain**

**Reviewer’s Comments**

***General comments***

**Content**

The authors are to be commended and thanked for addressing the suggestions and recommendations. The manuscript reads very well. I have no further comments regarding the content.

Well done to the authors on undertaking their thorough research and on preparing a very comprehensive, well-informed and timely manuscript on this important topic. The published article will be of interest to readers around the world!

**Copy editing**

The manuscript is ready for publication, following minor copy editing (points below).

1. p.6, line 148: Delete the comma after ‘… COVID-19’
2. p.7, line 168: Add the word ‘current’, i.e. to read ‘The current research …’
3. p.8, line 188: Add the word ‘period’ immediately before ‘(March 11 to June …)
4. p.10, line 209: Split last word into two, i.e. to read ‘… intercoder reliability’
5. p.10, line 209: I don’t think there is a need for the word ‘and’ between ‘double’ and ‘blind’ as this is usually written simply as ‘double blind’
6. p.11, line 324: Remove comma and second full stop after ‘prominent’
7. Table 6: Place the label (descriptor) on the line above the table, consistent with all other tables.
8. p.19, lines 359-60: After the colon, replace the commas with semi-colons, i.e. to read ‘real; …. ; …. ‘ and …’
9. p.23, line 454: Use a capital ‘T’ for the correct title of *The Lancet.*

All Tables containing numbers:

10. Line up the ones under ones, tens under tens and hundreds under hundreds in all columns; and
11. Remove brackets in the footnotes to the tables, i.e. remove brackets surrounding * and **.

Tables containing words:

12. Adjust either width of some columns or size of font to ensure that words are not split incorrectly (e.g. across two lines) and that commas appear immediately after the relevant word.

---------------------------------------
